# *“I will take part in the revolution with our people”*: a qualitative study of healthcare workers’ experiences of violence and resistance after the 2021 Myanmar coup d’etat

**DOI:** 10.1186/s13031-024-00611-7

**Published:** 2024-08-20

**Authors:** Rohini J. Haar, Katerina Crawford, Larissa Fast, Than Htut Win, Leonard Rubenstein, Karl Blanchet, Louis Lillywhite, Nicholus Tint-Zaw, Myo-Myo Mon

**Affiliations:** 1 University of California, Berkeley School of Public Health, Berkeley, CA USA; 2 RIAH Consortium., Stockholm, SE Sweden; 3 RIAH Project; Humanitarian and Conflict Response Institute, University of Manchester, Manchester, UK; 4 Independent researcher, Lincoln, NE USA; 5 Johns Hopkins Bloomberg School of Public Health, Baltimore, MD USA; 6 Faculty of Medicine, Geneva Centre of Humanitarian Studies, University of Geneva, Geneva, Switzerland; 7 Chatham House, London, UK; 8 RIAH Project, Graduate Institute of International and Development Studies, Geneva, Switzerland; 9 Freelance Researcher, Naypyidaw, Myanmar

**Keywords:** Myanmar, Civil disobedience Movement, Violence against healthcare, Attacks on health, Conflict, Healthcare workers, War crimes

## Abstract

**Background:**

In Myanmar, ongoing conflict since the 2021 military coup d’etat has been characterized by targeted violence against health workers (HWs), particularly those participating in the pro-democracy movement. Existing knowledge about the challenges faced by health workers in Myanmar is scant, including their perspectives on mitigating their suffering and the broader impact on community health. This knowledge gap prompted our study to assess the extent of the violence, its impact on the workers and the community, and identify resource priorities.

**Methods:**

This qualitative study employed purposive and snowball sampling to recruit health workers affiliated with the Civil Disobedience Movement (CDM). We interviewed 24 HWs in Myanmar between July and December 2022, predominantly physicians and nurses. We used a semi-structured interview guide and conducted interviews remotely due to the security situation. We adopted content analysis to understand participation in the CDM movement, experiences of violence, personal and professional impacts, the sequelae to community health, how HWs responded as well as their ongoing needs.

**Results:**

Thematic content analysis revealed that violence was both individually targeted and widespread. Health workers faced professional, financial, and personal impacts as a result. The health system as a whole has been severely diminished. Health workers have had to adapt to continue to provide care, for example some fled to rural areas and worked clandestinely, exchanging their services for food and shelter. In those settings, they continued to face insecurity from airstrikes and arrests. Health workers have also experienced moral distress and burden due to their resistance and protest against the regime.

**Conclusion:**

The coup and ensuing violence severely disrupted the healthcare system, resulting in shortages of supplies, reduced quality of care, and exacerbated challenges during the COVID-19 pandemic. Despite facing significant hardships, HWs remained resilient, engaging in resistance efforts within the CDM and seeking support from local communities and international organizations. They expressed a need for increased awareness, financial assistance, and concrete support for the health system to address the crisis.

## Background

Attacks on health during armed conflicts have intensified in the past twenty years as have efforts to document them [1]. Reports have detailed severe attacks on health facilities, such as airstrikes, and their direct consequences, such as effects on health facility operations, and the resulting deaths and injuries among personnel and patients. However, violence against healthcare may not always be high intensity: health workers are frequently arrested and detained, blocked from providing healthcare, and subject to bureaucratic persecution and unfair labor practices [2]. Over the past several years, civil society actors and scholars have repeatedly drawn attention to the broader and longer-term impacts of violence on health workers, the health system, and the communities they serve [3–10]. The situation in Myanmar since 2021 is striking, characterized by both targeted violence against healthcare workers (HWs) and widespread violence against health care facilities, each exerting significant and likely long-term impacts on health [11].

Myanmar, formerly known as Burma, has a complicated and often violent political history [12]. From 1962 to 2011, Myanmar was under military rule, characterized by suppression of dissent and repression of ethnic minority communities. A gradual liberalization process began in 2010, leading to free elections in 2015 and the formation of a civilian government under Aung San Suu Kyi in 2011 but the military remained powerful [13]. While many Myanmar nationals hoped for further reforms, persecution and violence including ethnic cleansing continued, combined with denial of labor rights, education and healthcare access, as well as the brutal ethnic cleaning of Rohingya people in 2017 [14, 15]. Elections in late 2020 resulted in a large majority vote for the civilian government, but the transition was thwarted by a military coup d’etat in February 2021. The coup, which was internationally condemned, led to widespread protests soon following [16]. The protest movement coalesced into the formation of a National Unity Government, composed of many ethnic minority groups, as well as senior positions for Aung San Suu Kyi and other civilian leaders. By May 5th, 2021, the National Unity government announced the formation of an armed wing, the People’s Defense Force (PDF) which marks the start of the current civil war in Myanmar, between the military regime and the National Unity government. The military junta-led regime, along with its armed counterpart, known as the Tatmadaw, has committed numerous violations of human rights, including violent suppression of protest, shutting down social and news media, forced disappearances, massacres, kidnappings, torture, extrajudicial detentions and executions and violence against civilians throughout this time [17–20].

The Civil Disobedience Movement (CDM) began in February 2021 as part of the protest movement [21]. HWs and many other civil servants across the country, including in the national capital, Naypyidaw, and largest city, Yangon, refused to work for or with the military regime and adopted a “no recognition, no participation” slogan against the military regime [22]. One expert on Myanmar’s civil service system estimated that approximately three-quarters of civil servants left their jobs at the height of the protests [23]. Among them, HWs from 110 hospitals and healthcare agencies initiated a labor strike that led to a rapid deterioration of health services [24]. They faced intimidation and threats from superiors [16]. By 9 February 2021, because of the lack of HWs, COVID-19 vaccinations had been suspended, the testing system had all but collapsed, and most hospitals in Myanmar had shut down [25, 26]. Many HWs have remained on strike since 2021 [26–28]. Due to the health sector’s significant impact and popular support among the population, the junta has arrested, threatened, and harassed CDM movement members. Several articles suggest that the crackdown resulted in a decline in and financial hardship for active members, and shifted public support towards the PDF rather than non-violent protest [29–31].

While the violence has affected many civilians, emerging reports from healthcare workers across the country suggest that they have been particularly targeted [17, 32–34]. This targeting of health workers in particular may result from the role of the health sector in demonstrations and political action against the military regime, its high representation in the CDM - ranking as the second highest in number of staff to have left government posts- and the positions of healthcare workers as leaders in the community, as well as their obligations to treat all patients based on well-established ethical codes and triage rules [35, 36]. Reports indicate that the military and police have targeted doctors and other HWs by arresting, injuring, and killing them as well as directly occupying medical facilities and raiding or physically damaging facilities and ambulances [33]. HWs have had their medical licenses revoked, been fired, or surveilled and targeted at their places of work [35, 37].

The incidence of attacks on health has been increasing, with a notable shift towards targeting health facilities rather than specific health workers. Since 2022, reports indicate the military regime is targeting hospitals in rural and opposition areas with airstrikes, aiming to destroy what little healthcare still exists [38–41]. Insecurity Insight, an international monitoring group, has documented over 1,325 attacks on Myanmar’s healthcare system in the more than three years from February 2021 to May 2024 [42].The number of such attacks tripled between 2022 and 2023. The majority of these incidents since 2022 have been concentrated in the northern and central regions surrounding the cities of Sagaing and Mandalay, where opposition groups’ resistance to the military regime is most pronounced [6]. In 2023 alone, there were 418 recorded incidents, 133 of which involved the damage or destruction of health facilities [6]. In response to the violence and the collapse of the healthcare system, some HWs have fled the country [43]. This has led some physicians and other HWs to work clandestinely—seeing patients in secret, moving to tribal areas, avoiding work in public places to circumvent being targeted, and utilizing social media for activism [44–46].

Beyond media and human rights reports, however, little is known about the specific issues that health workers within the CDM movement in Myanmar face, their views on how to improve the situation or their perceptions of the impacts of this persecution on the wider health of the community. This study addresses that gap, aiming to assess the extent and characteristics of the violence against HWs, the impact on the health system, and potential lessons on mitigation and protection based on the experiences of HWs. This qualitative study aimed to understand both the lived experiences of HWs in Myanmar and their perspectives on how the conflict is impacting the healthcare system and their communities [47].

## Methods

We used a semi-structured interview guide with open-ended questions to examine the lived experiences of HWs and their perspectives on impact. We adapted an existing interview guide for the Researching the Impact of Attacks on Health (RIAH) project to better account for conducting remote interviews and for the specific context of Myanmar [48, 49]. The interview guide asked about demographics, engagement with the CDM movement, and the impact on HWs’ personal and professional lives, as well as on the health system and community. We collaborated with researchers in the Myanmar diaspora and within Myanmar to conduct interviews in participants’ native languages. All interviewers were Myanmar nationals and healthcare professionals. Given the significant security risks that HWs face, we have obscured the identities, country of current residence, and gender of the interviewers. We conducted introductions and training sessions on Zoom with all researchers prior to conducting the interviews.

### Participant selection

This study utilized a purposive and snowball sampling approach to recruit HWs during the summer and fall of 2022. Given our focus, we only recruited HWs actively engaged or affiliated with the CDM movement. Due to the precarious security situation, the interviewers, led by NT, TH and MM, reached out to trusted communities within Myanmar’s health worker network to recruit participants. Those who were interested responded to the interviewers and could also forward messages to others in their network. Interviews were then scheduled and conducted remotely. We recruited participants living in any state and region within Myanmar because there was a great deal of displacement since the coup.

All participants were 18 or older, self-identified as health professionals (broadly defined to include doctor, nurse, midwife, health technician, pharmacist, dentist, other health professional or health administrator), had lived and worked in Myanmar for at least 2 months since February 2021, and had access to the encrypted Signal phone and messaging application to conduct the interviews. Signal was well-suited to interviews in this context because of its end-to-end encryption for both text and calls, screen security features, and minimal collection of sensitive data [50]. We particularly encouraged participation from women and non-physicians to enrich the sample. Given the nature of the study, external verification of credentials was not conducted. Audio- interviews were conducted remotely with all participants to ensure privacy and security. The sampling for the study was terminated once the interviewers assessed that we had reached saturation of ideas.

### Data collection and analysis

Due to security concerns for participants, we did not record interviews, nor store their names or identifying characteristics. Investigators took detailed notes in Burmese including transcribing quotations during the interview process whenever possible. These notes were stored in secure encrypted files and later translated to English by bilingual interpreters (NT and TH). English transcripts were uploaded to Dedoose Research Software Version 4.3. for qualitative analysis [51].

Interview questions focused on four major areas - demographic information, experience of attacks, engagement with the CDM movement, and impacts of attacks on the health system - with coding organized around these a priori domains. RH conducted the preliminary analysis by reviewing half of the transcripts and created an initial codebook that identified deductive themes from these domains and inductive ones from interview transcripts using a content analysis methodology [52, 53]. KC independently read all transcripts and developed additional, nested codes. RH then re-analyzed the transcripts, reviewing all codes and adding more where relevant. Following the initial independent analysis, the analysts discussed the resulting codes and emerging concepts and then grouped them into overarching themes. Any discrepancies were resolved by consensus.

### Ethical considerations

Ethical approval for this study was granted by the University of California (Protocol ID: 2022-01-14966). Given the nature of the study, assessment by a local institutional review board was not possible. Due to the ongoing armed conflict, the safety of both the participants and the interviewers (some of them in Myanmar) was paramount. More detailed security processes and precautions are available at https://github.com/rohinihaar/RIAH-.

## Results

We interviewed 24 participants between July and December 2022 who all lived in Myanmar at the time of the interview.

### Participant characteristics

Of the 24 people interviewed, 12 identified as male, eight as female, and four preferred not to answer. Their ages ranged from 23 to 45. Most were physicians (8/24, 33%) or nurses (7/24, 29%). Other HWs included dentists [2], midwives [2], public health supervisors [2], as well as a community health worker, health administrator, medical student (1 each); we did not ask about ethnicity. Demographic information about the interviewees can be found in Table 1. All HWs who reached out during the recruitment phase participated in the study and no participants stopped the interview before completion.

**Table 1:** Description of the interviewees

Demographic variable Category	*n* (%)
**Total Sample**	24 (100%)
**Sex**	
Male Female Not reported	12 (50%) 8 (33%) 4(16%)
**Age (years)**	
23–27 28–32 33–36 38–45	6 (25%) 8 (33%) 6 (25%) 4 (17%)
**Profession**	
Community Health Worker Dentist Health Administrator Medical student Midwife Nurse Physicians and Surgeons Public Health Supervisor	1 (4%) 2 (8%) 1 (4%) 1 (4%) 2 (8%) 7 (29%) 8 (33%) 2 (8%)

### Thematic analysis

The responses of the interviewees can be grouped into three themes: (a) experiences of violence; (b) perspectives on HW and community impacts; and (c) resistance and response.

- *Experiences of Violence*.

**Targeting Healthcare workers as individuals**: All participants described frequently facing threats and attacks or witnessing or hearing about colleagues being targeted directly. Participants described arrests, physical assault, being shot, sexual violence, and frequent threats. In a few cases, family members were also targeted or jailed, which participants described as efforts to coerce the HWs into stopping their clandestine work or surrendering to authorities. One participant described colleagues’ experience, *“My friends in [retracted town] got their houses burnt”* (Participant 10). Targeted arrests of health workers were common, especially early on. One respondent described their experience trying to avoid arrest: *“They came to our staff housing … to arrest CDMers. What we did at our housing was someone locked our housing from outside*,* and [we] pretended there was nobody inside”* (Participant 20).

Several respondents noted that some colleagues disappeared after detention and were not heard from again. According to one HW who was arrested for participating in the CDM movement, *“They got hit a lot*,* got broken hands*,* and head injury*,* and open wounds [on the] hands. They didn’t stitch the open wounds inside prison. They had to close the skin breaks (open wounds) after they had arrived at their home. They said they were still suffering from headaches due to having their head beaten inside”* (Participant 1).

Sixteen interviewees mentioned physical assault and beatings from both personal experiences and secondhand knowledge. Twenty-one of the respondents mentioned HW arrests during or following a protest in the months after the coup and after providing medical care to anyone accused of being against the military regime. One HW said; *“The protest group I was involved in was attacked on March 6th*,* 2021. They surrounded us from two opposite sides to arrest us. I got beaten by a baton. They pulled the hair of our protecting line members and beat their backs. I think they did [that] because we were Healthcare workers wearing uniforms”* (*Participant 24).* While many civilian protestors were victims of attacks; respondents reported that HWs were specifically targeted by military forces. One HW discussed the result of this targeting, *“…I was a former member of [the] University of Medicine student union. My name was on their list. Thus*,* I had to flee from home since March 21*,* 2021”* (*Participant 2).* HWs were often involved in protests against the military regime and provided care to other protestors at great personal risk. At other times, because of the risk to their lives, they tried to support colleagues remotely but felt a great moral burden. One participant said that in one protest, *“The actual bullet was passing just above my head” (Participant 15).* As a result, the participant feared to go out again, stating that *“Since I couldn’t go there*,* I had to help them by giving instructions by phone. We couldn’t go out to the streets. If we did*,* we would be shot dead” (Participant 15).*

Firearms, using live ammunition and rubber bullets, were commonly used against HWs and protestors according to 20 respondents. One HW described their experience: *“They used real bullets. I saw the bullets pierce the helmets. I even witnessed a healthcare worker-motorcyclist [being] shot in front of me. I had to remove the bullet pieces at thigh*,* and arms. I remember some lost hands*,* some lost legs…and also half of the face. One of my patients was [a] 40-year-old lady injured by SAC’s [State Administration Council] bombing”* (Participant 6).

Many of the interviewees had participated in the initial phase of protest marches and were victims of crowd control weapons in that period, including sound and water cannons, and tear gas, as well as kinetic impact projectiles (rubber bullets). One participant noted of that period, *“Of course*,* I was the victim of violence. When it was for [retracted march name]*,* tear gas and sound bombs were shot at us. I also got them (tear gas canisters) when I was involved in protests like in [retracted] township”* (Participant 1).

Seven interviewees described hearing about the use of sexual violence from their networks. *“The military committed sexual violence such as handling female genitals by hand*,* penetrating female organs by sticks*,* and standing upon the male genitals with pressure by military boots”* (Participant 2). Another participant described what happened to a female colleagues: *“a female doctor [was] mistakenly arrested by them…[she was] also had beaten*,* kicked nearly to death*,* like lost teeth*,* and got bruised over her whole body*,* and also got sexual violence”* (Participant 1).

Participants also noted surveillance, by both the police and military, and both implicit and explicit threats as methods of causing psychological distress, focused on HW involvement in the CDM movement. One HW described receiving calls and threatening messages in this way: *“the police came to that clinic not wearing uniforms*,* took photos*,* and we knew that we were [being] watched”* (Participant 9).

**Targeting facilities and transports**: Healthcare facilities and healthcare transports experienced aerial and ground assaults by the State Administration Council forces (SAC, Myanmar’s ruling military junta), especially since mid-2021. One interviewee described creating mobile clinics because the existing hospitals were not safe: *“…the military bombed the temporary clinics and hospitals in [retracted location] with aircrafts. The Healthcare workers*,* including doctors and nurses*,* had to flee from it”* (Participant 2). Attacks on facilities took many other forms including destruction or seizure of health centers, closure of facilities, blockades, and theft of medical equipment. Several participants said officers would enter the hospital at night to beat and arrest HWs, break oxygen machines, target patients, occupy the facility, and generally render it unusable. One participant said, *“They forced [us] to close the pharmacies*,* and punished the people that lined [up] for drugs. They close[d] the oxygen resources by destroying them”* (Participant 4). This was especially notable given that this violence was taking place in early 2021, when the COVID-19 pandemic was taking a large toll on the region. Often these various forms of violence that were perpetrated by SAC forces occurred in conjunction with each other with the ostensible goal of tightening control over the entire health sector. One participant described the severity of the control: *“SAC wouldn’t let [us] carry drugs without their approval letters*,* healthcare service is so so poor. Even basic essential health services couldn’t be delivered…”(Participant 20*). The military attacked and seized healthcare transport, particularly ambulances. One nurse described a personal experience of being detained following an attack on their ambulance.

**Denial of healthcare to the community**: Beyond targeting CDM-affiliated HWs, the SAC also denied healthcare to certain groups of patients, particularly those who supported the opposition movement and those from rural, ethnic areas. HW interviewees mentioned examples of this behavior, including providing COVID-19 vaccinations only to SAC supporters, punishing people for standing in line for medication, and destroying medicine or equipment. One interviewee described how the military confiscated equipment: “*To carry drugs*,* like RDT (rapid diagnostic test) kits for malaria were blocked and taken by the military”* (Participant 20). Participants reported that the SAC intentionally lowered the quality of care or denied access to healthcare for those who opposed the regime. One HW discussed their experience, *“the public hospitals only accepted the people that are friendly to non-CDM. They denied others. [The] husband of my wife’s sister had pain in [his] abdomen and went to hospital*,* he was denied to even enter to the hospital. They tested him [for] COVID in his car. They referred him to [private hospital] since he tested positive. The hospital didn’t take care of him*,* nothing”* (Participant 19).

b) *Perspectives on HW and community impacts*.

The various forms of violence perpetrated against HWs led to injury, death, and other long-term impacts. Of the 24 respondents, 13 mentioned the death of HWs, non-HWs, and other protestors either as a direct result of attacks by the military (i.e. weapon use) or as a secondary result (i.e. death of patients from COVID-19 due to the confiscation of oxygen cylinders from clinics). In several cases, HWs were killed while attempting to treat patients. Injuries among HWs and other protestors were also frequently described. One HW described their memories of a protest, *“I face violence at [location] protest. I felt endangered for my life. I heard gunshot sounds. I saw a girl get a superficial gunshot wound at her ear”* (Participant 17).

#### HW impacts

The coup and subsequent violence impacted all facets of life in Myanmar for all residents. For HWs who actively opposed the SAC, these included professional, financial and personal/ family impacts. In particular, participants described both physical and psychological disabilities resulting from the violence they and their colleagues experienced. Some colleagues became permanently disabled, some lost limbs, others suffered from wounds that took months to heal, and some had psychological trauma as a result of arrests and torture. *“A doctor friend of mine came out of prison after staying for five months there… and I found that they became so afraid of everything. Like not in sanity”* (Participant 1).

For many HW interviewees, their involvement in the CDM movement meant losing their jobs in the healthcare field and being blacklisted. Many indicated that clinics were unable to legally hire them because of their CDM affiliation. Moreover, many CDM-affiliated HWs could no longer practice medicine in public, causing them to flee to rural areas or to practice in secret. One HW described their experience:*“[I] came back to my hometown*,* became jobless*,* depressed*,* and most private hospitals wouldn’t accept me to hire since I am CDM. Not even NGOs. Even if they accept CDMs*,* they reduce allowances. I never went back to [the] hospital” (Participant 10).* Several interviewees adapted to the hostile environment and continued to service their patients through online consultations and volunteering at charity clinics.

HW interviewees experienced serious financial hardship because they were forced out of or unable to work, compounded by the effect of rising inflation. The situation was particularly dire for the primary income-earners who supported their immediate and extended families. One HW stated, *“I couldn’t support my home. It becomes more destitute. I couldn’t get any other jobs since I am a CDM person”* (Participant 17). Another said without their income for the extended family, everyone was *“worried and afraid”* (Participant 5). One participant described the burden of disappointing parents and children: *“I had to drop my parents’ hope. I am now struggling for my family and my children’s education. All the people that are taking part in revolution are not safe*,* including my family. [I am] endangering our lives”* (Participant 3).

Several participants described the challenge of managing living conditions. For some, the compounded stress of violence, finances and occupational security was overwhelming. One HW stated, *“It is unsure that I would be arrested or not if I go back home since I am currently in the jungle now. I couldn’t sleep well during the nights. I have to say stand-by even here*,* worrying when I would run away”* (Participant 23). Another discussed the emotional toll: *“I don’t want to think about the future… It is so disappointing. [My] future plans are totally ruined”* (Participant 1). Some interviewees noted their inability to sleep, feelings of stress, and a sense of uncertainty for the future. Many relayed feelings such as *“I feel insecure always*,* and mostly at night….Now*,* everything is upside down*,* nothing sure. I even feel like enough to stop nursing work after this”* (Participant 13).

Beyond the financial hardships inflicted upon HWs and their families, several interviewees described feelings of fear and insecurity as well as the loss of many relationships due to their CDM affiliations. As one HW said, *“There is a huge divide between CDM and non-CDM among close friends. They end their friendships like they don’t talk to each other. I also had some friendships ended with some teachers…I need to be aware of the environment …. it is very risky in security* “ (Participant 15). As several HWs stated, the possibility existed that previous colleagues who were not part of CDM would report those who were part of the movement. One participant warned, *“There is neither social safety nor personal safety since I was trying to be aware of who would stab my back. I needed to stay very cautious”* (Participant 15). *A*nother noted the sadness of losing friends, *“Everyone is affected. Friendships were broken”* (Participant 16).

Several participants touched on the moral injury of working in this context (the distress that results from professionals facing ethical dilemmas, such as prioritizing limited resources or witnessing suffering). On the one hand, as health workers, they had a professional duty to care for the sick. On the other hand, they felt that working under the military regime violated their obligations to the nation as citizens. A few rejected the idea of treating police or military members while others acknowledged their medical obligations to treat everyone. Similarly, they felt solidarity with other colleagues within the CDM movement while also feeling the pressure of needing employment and an income to keep their families fed. These tensions mounted over months and years, causing ongoing distress. Some HWs returned to work under the military regime while others received support from local NGOs, and some became destitute. One HW described it in this way: *“The worst is being destitute… I know the coup is not fair. But I didn’t have enough courage to leave my work… it’s like having a dilemma”* (Participant 19). Some HWs noted a loss of dignity and future while others reaffirmed their commitment to the cause and underscored their belief that, in the end, they will be victorious against the regime.

Most interviewees described their families as being supportive of the CDM cause, and indicated siblings or family members also joined the movement. Others, however, became estranged from family members who did not agree with their decision to be part of the movement. One HW noted, *“My household members didn’t say much to me. The main thing was that I couldn’t support them anymore*,* as I used to do by my outside job. That’s it”* (Participant 1). The tension between supporting the resistance movement and family was intense, including one who said, *“We are doing CDM and my parents [are] worried whenever we are traveling. I would like to go back to [hometown] to collect my belongings*,* but my family didn’t allow me to do that because they are afraid that I would easily be arrested”* (Participant 20).

**Community and health system impacts**: All HWs interviewed discussed the detrimental effects of the coup and the violence against health on the healthcare system, including the lack of medical supplies, the reduction in the quality of care, and the long-term impacts for patients and overall population health. Regarding wider societal impacts, one HW forecasted that, *“All will be ruined. I mean*,* the healthcare system. For sure*,* Myanmar will be ranked as 192nd country if there are 191 countries in the world”* (Participant 8). One HW described the impact of equipment shortages and the task shifting that is now required: *“It is very different from now and then*,* in treating patients. There were many doctors and drugs around me at [the] hospital. Now*,* everything is limited*,* like medicine*,* and also*,* I have to manage myself. That’s why I am struggling in some procedures that [are] beyond my expertise”* (Participant 22). Shortages in medicine and staffing forced some patients to suffer at home or only seek medical help in dire situations. *“Even in-labor patients stopped going to the hospital since they knew they wouldn’t get any service from the hospital”* (Participant 23). The resource scarcity resulted in more strife, *“Oxygen cylinders were kidnapped*,* and taken since they were in high demand. Some died even though they shouldn’t have died. They were very young”* (Participant 18).

Primary care was particularly affected by attacks on facilities and the flight of HWs and many areas stopped providing basic services such as routine immunizations. One participant noted, “*there were …dysfunctional [outpatient] services too. Not even functional to half of the capacity before*” (Participant 23). This was particularly concerning as tuberculosis (TB) has been a serious issue in the country. Another HW lamented, *“…TB patients hardly got their drugs*,* and had to stop taking their medicine. I am very stress[ed] about those mainly. I just want drugs for them. If not*,* [they] will develop drug resistant TB” (Participant 13).* Childhood vaccination campaigns and other preventive care services were reduced or stopped following the coup. One participant described that *“Routine Immunizations like EPI (childhood vaccination programs) couldn’t be run…Areas that cannot run EPI for over a year couldn’t have any basic health service. No primary healthcare too. Not systematically [anyway]”* (Participant 20).

The COVID-19 pandemic exacerbated the problems faced by the health system following the coup. One participant described that *“fear of bullets is more concern[ing] than preventing COVID”* (Participant 2). COVID-19 vaccination campaigns, previously conducted with some success, either no longer occurred or were confined to certain parts of the country (particularly pro-military regime hospitals). Many interviewees described excess deaths from COVID-19 due to lack of preventive vaccines as well as basic treatments including oxygen.

These service challenges were by no means homogenous across the country. Inequality between rural and urban areas became apparent following the coup to the participants, since some services were unavailable in rural villages. Several HWs explained that some military-regime run hospitals shut down while private hospitals charged high rates, effectively denying access to people who could not afford the cost, were ethnic minorities, or affiliated with the CDM movement. One HW described the decline in availability over time: *“At first*,* [wealthier] people went to private clinics*,* but they eventually were full. Then*,* people couldn’t get service even if they could afford it”* (Participant 15).

c) *Resistance and Response*.

All HWs interviewed were involved in some form of resistance within the Civil Disobedience Movement. Many HWs stated that they attended protests against the military and junta military regime both as activists and to provide medical care for others as street medics.

Several described their CDM role as resisting the coup military-regime. They wanted to support the revolution and opposed working in a military-led health system, since it undermined health: *“doing CDM doesn’t mean not willing to serve the people but being disobedient to military junta and coup. Therefore*,* I have no intention to go back to work during their era”* (Participant 4). For some, the choice to join CDM was right and a source of pride. One HW described this sentiment: *“I did CDM to destroy [the] military junta’s system. I will go back to work after the revolution has prevailed”* (Participant 5).

In the context of targeted violence and the hardships of working clandestinely, HWs in Myanmar adapted to life under the regime in order to maintain their resistance efforts. Some activities, such as collaborating with non-military regime organizations, developing a community with other CDM professionals, and maintaining lines of communication via social media, supported a sense of solidarity. In some cases, local nonprofits provided financial support, protection or resources to people caring for their communities. One respondent described this mutually beneficial relationship: the *“clinic was already opened before that. It is [an] NGO clinic. We just joined them as they welcomed us. We just need to contribute manpower and drugs”* (Participant 9). In some cases, the CDM network, local health organizations or international NGOs, and charities provided supplies such as oxygen machines, or medications that facilitated basic medical care. In exchange, HWs were housed by local village people, *“I am now fed by village people around here and the PDF (the armed forces of the National Unity Government)”* (Participant 4). One HW expressed their gratitude: *“I need to say thank you…I am very grateful to the people that came here to provide food for us taking [a] hard and long journey” (*Participant 3). A few participants described staying within the village as safer than living in a medical compound where they felt more vulnerable to arrest or attacks. According to one HW, *“I had to sleep over [at] other people’s houses. After all*,* I asked for help to [local group] and they provided a place to live. I feel safer there”* (Participant 13).

Perhaps because of the displacement, social media and internet connections were critical to maintaining links to families and communities, and as a way of spreading awareness and information despite severe restrictions on free speech and press. Many interviewees described seeing social media reports of other HWs beaten and killed by SAC forces, which they said compelled them to continue their resistance. However, they perceived social media, and Facebook especially, as a dangerous platform. While some used it as a way of understanding the sentiments and affiliations of coworkers and friends, social media fractured social circles and enabled surveillance. For example, one participant said, *“Everyone is affected. Friendships were broken. Blocking in social media was obvious”* (Participant 1). Another described that *“A friend of mine was warranted and arrested. They checked their phone and arrested [them] because of their Facebook posts. Now they are imprisoned”* (Participant 10). Private messenger groups were also regarded as a way to connect with friends and allies, share experiences, but carried a risk of being identified and arrested.

Several participants stated that they wanted others to know about the current situation in Myanmar, how they are feeling, and what people both inside and outside Myanmar could do to help. Many participants described their destitution and need for support - political, practical and financial (e.g. secure jobs and basic salary)- to achieve any meaningful protection. Others asked those inside Myanmar who have not yet joined CDM to take part in the cause. Several HWs emphasized their hopes that this study could amplify their voices and lead to tangible results, such as more charitable donations and resources to their underfunded clinics, as well as protections from military air and ground strikes. Above all, they want peace. One HW relayed this message, *“Please tell the world…that [a lot of] money*,* medicine*,* medical equipment and manpower are needed in our revolution”* (Participant 6).

## Discussion

This study sheds light on the challenges health workers in Myanmar face following the 2021 military coup d’état. The thematic analysis revealed that: [1] HWs in Myanmar, particularly those affiliated with the CDM resistance movement, face myriad attacks on and interferences with their work by the military regime, ranging from individual arrests to more and more, large scale airstrikes against health facilities; [2] this violence deeply affect health workers professionally, financially and personally; [3] the impacts on the health system and wider community are wide-ranging and point to the potential for longer-term health system catastrophe; and [4] that while HWs have survived through innovation and adaptation, they continue to live in dire circumstances. Finally, [5] this study of opposition-affiliated HWs illustrates the significant burden and moral distress that results from protesting against a powerful military regime and the costs of resistance.

One of the most distressing findings is the prevalence of targeted violence against HWs, including arrests, physical assault, sexual violence, and threats. The use of firearms, heavy weapons, and other forms of violence have instilled fear and resulted in injuries, deaths, and psychological trauma among HWs. Such violence not only violates the fundamental rights of HWs but also undermines the provision of healthcare services, jeopardizing the long-term health and well-being of the population. While attacks on healthcare workers are tragically common, participant testimonies paint a dire picture of targeted attacks aimed at suppressing dissent and maintaining control over the healthcare system [1, 6, 8, 54]. Although this feature is not unique to Myanmar, and targeted violence against HWs occurs in other conflicts, it is noteworthy in this context [55]. This finding is consistent with recent reports suggesting that while not frequently reported in media, Myanmar had the second greatest number of attacks on health in an armed conflict context globally in 2023 [6].

Despite facing grave risks, all HWs interviewed were proactively involved in the resistance movement, demonstrating their commitment to opposing the military regime. HW participation in civil disobedience and protests underscores their determination to uphold democratic principles. However, this resistance comes with a significant cost. Many HWs have experienced professional repercussions, financial hardship, and strained relationships with colleagues and family members. The loss of livelihoods, displacement, and isolation that result from their decision to join the CDM movement further exacerbate the challenges they face.

By withholding medical care from certain groups and targeting HWs affiliated with the resistance movement, the military junta not only exacerbates the suffering of the population but also undermines the integrity of the healthcare system. The deliberate destruction of medical facilities and equipment further compounds the humanitarian crisis, leaving communities without essential healthcare services, which is a violation of both human rights and International Humanitarian Law [56, 57]. Participants suggested that this damage is not homogenous, rural and already marginalized ethnicities within Myanmar are facing the worst health system impacts: with an already fragile health system and persecution of their health workers, this new assault has resulted in even less access. This disparity spans previous research in Myanmar (pre-coup) as well as ongoing media reporting [46, 58].

In response to these challenges, HWs have demonstrated resilience and resourcefulness in their efforts to mitigate the impact of the coup on healthcare delivery. Collaborating with non-governmental organizations, establishing community networks, and leveraging social media platforms have emerged as vital strategies for maintaining essential healthcare services and creating solidarity during the ongoing crisis. Despite these efforts, they continue to live under heavy security threats and frequently, in poverty. Supporting these health workers is a critical role for the international community, both with financial support, potentially via local organizations, and by calling attention to their experiences.

Finally, the study particularly highlights HW experiences of moral distress and its connection to resistance. Several respondents framed their CDM affiliation, at least initially, as their response to the military junta’s treatment of health services as another military branch [59]. Even as the coup was just beginning, participants noted that the junta wanted to upend triage priorities and ensure that they and their allies received first priority, regardless of medical need. Joining the CDM movement and the labor strike was, in their view, a first act of resistance to the interference with healthcare. At first glance, the moral distress participants expressed parallels that of HWs in other conflict settings [7]: the best care for the patient clashes with administrative and logistical hurdles, resulting in HWs feeling the burden of knowing how to help but not being unable to provide care. In this study, however, two additional dimensions of their moral distress are integrally linked to their resistance activities. First, they noted the tension between their duty to provide healthcare services for their communities and their duty to protest military rule for their country. Many also felt that their patriotic and ethical obligation to leave their roles in military-controlled hospitals conflicted with their need to provide for their families as the primary wage earner. The cost of resistance is significant, and similar to the costs for HWs in other conflict settings who chose to provide care for protestors, resulting in being targeted, arrested, jobless and often frustrated [60]. These findings represent an interesting contrast with our partner study in Nepal where health workers remained generally politically neutral through that long conflict. In that setting, the neutrality may have made the health system function through the conflict but it is not clear the same would happen in Myanmar given the vastly different conflict dynamics [10].

Health workers in Myanmar, and around the world, face a pressing dilemma: whether to abstain from or actively engage in civilian resistance movements against societal injustices and violent regimes. This decision is deeply individual and depends on local contexts, family situations, professional, social and emotional pressures, and personal convictions. The challenges faced by health workers engaged in the CDM movement starkly illustrate the multiple layers of both individual and systemic costs of active participation on the side of resistance. In the case of Myanmar, these include targeted and severe violence by the military against health workers and patients, frequently resulting in both direct and indirect health system destabilization and worsening morbidity, as well as moral injury, family pressures, and tensions around obligations to best care for the community.

Considering the violence and these impacts, both solidarity and meaningful support are critical for Myanmar’s health workers. Specifically, the international community is crucial in providing financial support via direct and indirect aid mechanisms to buttress the health work that is being done. Although some assistance is in place, it falls far short of addressing the enormous needs arising from the conflict and subsequent health system collapse [61]. Additionally, strengthening advocacy could include continuing to document the violence and bear witness to the impacts, as well as pressuring the military regime to respect democratic principles and restore protections for healthcare. Ongoing research urgently needs more support, especially for scholars based in Myanmar to lead the work and for enhanced partnerships and cross-disciplinary collaboration focused on prioritizing limited resources.

### Limitations

This study has several important limitations. With remote purposeful and snowball sampling and a sample size of 24, we were able to gain valuable insights into the range of attacks and impacts experienced in a very complex and insecure context. However, this study is not a representative sample of health workers in Myanmar or even those working with or sympathetic to the CDM. We may have limited access to some HWs, for instance those without internet access, from more remote ethnic areas, or who are less technologically adept. Our study skews towards younger health workers, suggesting that there may be a need to sample more established workers as well. For this paper, we did not seek the perspectives of non-CDM healthcare workers or those who left the country following the coup; their experiences with conflict are also important. Since we only spoke with health care workers affiliated with the CDM, it is possible that some answers may have been biased based on their political views. Given the dynamic and insecure context, we were only able to conduct remote interviews. With in-person access to health workers in Myanmar if the situation stabilizes, we would be better able to bear witness to their day-to-day experiences, have more in-depth contextual understanding of their responses, and develop a stronger rapport. This study also has unique strengths: we recruited health workers from different regions, professional backgrounds and genders while working with a trusted local network. These local workers were able to interview participants in their local languages and allow them to speak unguardedly despite the incredible risks. While this exploratory study touches on issues facing Myanmar’s health workers, more research will be necessary to understand the scope and scale of these issues and assess interventions to mitigate them.

## Conclusions

Since the 2021 military coup d’état in Myanmar, health workers have been under increasing pressure, and many have been subject to violence, targeting both individuals and the broader health system infrastructure. Through this study, we were able to learn more about the experiences of health workers in the CDM movement in Myanmar: [1] attacks and interference by the military regime include arrests, airstrikes and even health facility bombings, [2] professional, financial and personal burdens on HWs are exacerbated due to that violence, [3] health system impacts mount and may lead to collapse, [4] while HWs are resilient, living conditions remain dire, [5] ethical dilemmas arise as a result of resistance and protest. Health workers have adapted to provide care to communities under these conditions; however, support is needed to avoid further decimation of the healthcare system. Health workers interviewed emphasized the need for humanitarian and financial assistance as well as international awareness-building to protect and bolster the healthcare system to help themselves and the greater civilian population.

## Data Availability

No datasets were generated or analysed during the current study.

## Abbreviations

CDM: Civil Disobedience Movement
HW: Healthcare worker
PDF: People’s Defense Force
SAC: State Administration Council
